# An Optimal Radial Basis Function Neural Network Enhanced Adaptive Robust Kalman Filter for GNSS/INS Integrated Systems in Complex Urban Areas

**DOI:** 10.3390/s18093091

**Published:** 2018-09-13

**Authors:** Yipeng Ning, Jian Wang, Houzeng Han, Xinglong Tan, Tianjun Liu

**Affiliations:** 1 NASG Key Laboratory for Land Environment and Disaster Monitoring, China University of Mining and Technology (CUMT), Xuzhou 221116, China; 3891197ning@163.com (Y.N.); tjliu_cumt@126.com (T.L.); 2 School of Environment Science and Spatial Informatics, China University of Mining and Technology (CUMT), Xuzhou 221116, China; 3 School of Geomatics and Urban Spatial Informatics, Beijing University of Civil Engineering and Architecture, Beijing 100044, China; hanhouzeng@cumt.edu.cn; 4 School of Geodesy and Geomatics, Jiangsu Normal University, Xuzhou 221116, China; tanxinglong3@126.com

**Keywords:** GNSS/INS, dynamical model error, observation gross error, fault detection, optimal RBF neural network, adaptive robust filtering

## Abstract

Inertial Navigation System (INS) is often combined with Global Navigation Satellite System (GNSS) to increase the positioning accuracy and continuity. In complex urban environments, GNSS/INS integrated systems suffer not only from dynamical model errors but also GNSS observation gross errors. However, it is hard to distinguish dynamical model errors from observation gross errors because the observation residuals are affected by both of them in a loosely-coupled integrated navigation system. In this research, an optimal Radial Basis Function (RBF) neural network-enhanced adaptive robust Kalman filter (KF) method is proposed to isolate and mitigate the influence of the two types of errors. In the proposed method, firstly a test statistic based on Mahalanobis distance is treated as judging index to achieve fault detection. Then, an optimal RBF neural network strategy is trained on-line by the optimality principle. The network’s output will bring benefits in recognizing the above two kinds of filtering fault and the system is able to choose a robust or adaptive Kalman filtering method autonomously. A field vehicle test in urban areas with a low-cost GNSS/INS integrated system indicates that two types of errors simulated in complex urban areas have been detected, distinguished and eliminated with the proposed scheme, success rate reached up to 92%. In particular, we also find that the novel neural network strategy can improve the overall position accuracy during GNSS signal short-term outages.

## 1. Introduction

GNSS is a commonly used technology in location-based services (LBS). A typical land vehicle navigation system (LVNS) based on GNSS has to operate in dense urban areas where GNSS signals are either blocked or severely degraded by phenomena such as cycle slips or multipath effects, which limit its capability to achieve satisfactory accuracy and positioning reliability [1]. An Inertial Navigation System (INS) can provide continuous position information and accurate attitude information during GNSS signal failure [2]. It is clear that integrating GNSS and INS can deliver an enhanced performance compared to the individual systems [3]. Recently, advances in micro-electro-mechanical-systems (MEMS) inertial sensor technology have rendered the integrated GPS/MEMS INS system an attractive low-cost option for positioning in an LVNS [1].

Kalman filtering has been applied for many years to provide an optimal GPS/INS integrated module [4,5,6]. However, on the one hand, the system performance of a GNSS/INS integrated LVNS entering a tunnel, a downtown area with high buildings, a canyon or a forest may frequently be degrade, bringing gross observation errors [4] into filtering. INS will drift swiftly out of its planned trajectory over time so that the vehicle may become lost during a mission, especially when a low-cost MEMS inertial measurement unit (IMU) is used. On the other hand, MEMS IMU outputs are corrupted with high noise and large uncertainties, such as bias, scale factor and non-orthogonalities [5]. Meanwhile, dynamic inconsistency between vehicle and sensors also lead to IMU gross errors [6]. Unlike observation gross errors, this case may bring dramatic dynamic model errors into filtering so the estimation is very different from the real dynamic system.

As a consequence, fault detection and isolation (FDI) techniques have become necessary to cope with these two types of errors [7,8]. Actually, it is hard to distinguish dynamical model errors from observation gross errors using observation and state residuals because the residuals are affected by both dynamical model errors and observation gross errors in loosely-coupled integrated navigation system [9]. Most adaptive or robust KF based on state chi-square test and residual chi-square test can only restrict one of them effectively [10,11]. Recently, due to the powerful ability of dealing with nonlinear problems, several techniques based on neural networks (NN) have been proposed to replace KF in order to solve some of its shortcomings [12,13,14,15,16,17]. We can obtain optimal estimations of dynamic model for INS and GNSS data fusion when the dynamic model has significant errors if we build a reasonable neural network model [12], and it is helpful to localize the measurement outliers. Input-Delayed Neural Networks (IDNN) [13], Radial Basis Function Neural Networks (RBFNN) [14,15], Adaptive Neuron-Fuzzy Inference Systems (ANFIS) [16], Fuzzy Neural Network (FNN) [17], Multi-Layer Perceptron (MLP) network [18] etc., were all reported for GNSS/INS integration recently. The main advantage of RBFNN lies in its simple and fast learning rule, which in contrast to back propagation networks, is not iterative and has no convergence problems [19], which makes it more suitable for application in real-time processing systems.

In more recent studies, Jiang et al. [20] proposed an adaptively-robust strategy for GPS/INS integrated navigation systems to resist model deviations and outliers, but it applied only to tiny state perturbations and treated model deviations and outliers uniformly. Yao et al. [18] applied an improved Multi-Layer Perceptron (MLP) network to predict and estimate a pseudo-GPS position when the GPS signal is unavailable and demonstrated that proposed model can effectively provide corrections to standalone INS during 300 s GPS outages. Tian et al. [21] utilized improved RBFNN to predict INS errors during GPS outages. Navidi et al. [22] employed adaptive fuzzy inference systems (AFIS) aided KF algorithm to fuse low-cost IMU and GPS robust measurements. Generally speaking, most studies are focused on filtering robustness and estimation correction during GNSS outages with artificial intelligence algorithms. However, there is limited research regarding the GNSS/INS integrated system fault detection and recognition (FDR) with intelligent algorithms. 

This paper aims at introducing a novel optimal RBF neural network-enhanced low-cost GNSS/IMU system integration approach for FDR providing highly accurate corrections to the standalone INS during GNSS outages in complex urban areas. Firstly, we designed an optimal RBF neural network based on the optimality principle and sliding window strategy. During the “GNSS on” periods, our system performs navigation based on the integration of GNSS/IMU over a standard Extended Kalman filter (EKF). Meanwhile, the optimal RBF neural network will be trained with a sliding window. The output of the network can serve as a check value for observation outlier identification and isolation. Then a fault detection method based on Mahalanobis distance is put forward, whereafter robust and adaptive filtering algorithms, are proposed to reduce observation and dynamic model errors. In this way, the system can detect system faults and choose robust or adaptive Kalman filtering autonomously for the purpose of adjusting the contribution of observations and dynamical models to the navigation result. Lastly, in the absence of GNSS signals, this model operates in the prediction mode to generate and supply the estimated GNSS position difference data to prevent the vehicle from leaving its path. In order to verify the effectiveness of the proposed method, a GPS/BDS real-time kinematic (RTK)/MEMS IMU integrated hardware and software have been developed, then a land vehicle test has been performed.

The structure of this paper is organized as follows: the GPS/BDS/INS integrated navigation model is clearly described in Section 2. The optimal RBF neural network aided navigation fault detection, recognition and reduction technology are discussed in Section 3. Experiments and results of the proposed method are illustrated in Section 4. Finally, Section 5 draws the conclusions and ends the paper.

## 2. GPS/BDS/INS Integrated Navigation Model

In this research, a Loosely Coupled (LC) EKF is applied for GPS/BDS/INS integration. A 24-states EKF [23,24] is used for describe the system state, which can be can be described by Equations (1):

(1) $$\left\{ \begin{array}{l} X_{\mathrm{nav}}=[\delta r_{N},\delta r_{E},\delta r_{D},\delta v_{N},\delta v_{E},\delta v_{D},\\ \;\;\;\delta \psi_{N},\delta \psi_{E},\delta \psi_{D}{]}^{T}\\ X_{\mathrm{acc}}={\left[\nabla_{bx},\nabla_{by},\nabla_{bz},\nabla_{fx},\nabla_{fy},\nabla_{fz}\right]}^{T}\\ X_{\mathrm{gyro}}={\left[\varepsilon_{bx},\varepsilon_{by},\varepsilon_{bz}\right]}^{T}\\ X_{\mathrm{ant}}={\left[\delta L_{bx},\delta L_{by},\delta L_{bz}\right]}^{T}\\ X_{\mathrm{grav}}={\left[\delta g_{N},\delta g_{E},\delta g_{D}\right]}^{T} \end{array}\right.$$

where $X_{\mathrm{nav}}$ are nine navigation solution errors of three dimensional position, velocity and attitude in the north-east-down navigation frame (n-); $X_{\mathrm{acc}}$ are six accelerometer error modeling parameters (bias and scale factors for each axis) in the body frame (b-), $X_{\mathrm{gyro}}$ are three gyro drifts in b-, $X_{\mathrm{ant}}$ are three lever arm errors in b-, respectively; $X_{\mathrm{grav}}$ are three gravity uncertainty errors in the n- frame.

The discrete-time form of the dynamic model of the system has the following form:

(2) $$X_{k}=\Phi_{k,k-1}X_{k-1}+w_{k-1}$$

where $X_{k}$ and $X_{k-1}$ are the state vector at epoch *k* and *k* − 1, respectively; $\Phi_{k,k-1}$ is the state transition matrix (see [25]) from epoch *k* − 1 to *k*; $w_{k-1}$ is uncorrelated white Gaussian noise sequences. $w_{k}$ should satisfy the Equations (3):

(3) $$\begin{array}{l} E\{w_{k}\}=0\\ E\{w_{k}w_{i}^{T}\}=\left\{ \begin{array}{l} Q_{k}\;i=k\\ 0\;\;\;i\neq k \end{array}\right. \end{array}$$

where *E*{·} denotes the expectation function. $Q_{k}$ is the covariance matrix of process noise.

The antenna phase center in Earth Centered Earth Fixed (ECEF) (e-) frame in consideration of the deviation between the antenna phase center and the INS reference center can be written as:

(4) $$r_{\mathrm{GNSS}}=r_{\mathrm{INS}}+C_{b}^{e}L_{b}$$

where $C_{b}^{n}$ is the rotation matrix from b- frame to e- frame.

In this paper, the difference in position between GNSS measurements and INS measurements in the n-frame is regarded as measurements, so the integrated navigation observation model can be written as:

(5) $$Z_{k}=\left[\begin{array}{c} v_{\mathrm{INS}}^{n}-v_{\mathrm{GNSS}}^{n}\\ p_{\mathrm{INS}}-p_{\mathrm{GNSS}} \end{array}\right]=HX_{k}+V_{k}$$

where $Z_{k}$ is the observation vector at epoch *k*, ***H*** is the measurement matrix with the following form, $V_{k}$ represents the measurement noise vector:

(6) $$H=\left[\begin{array}{cccccc} 0_{3\times 3} & I_{3\times 3} & 0_{3\times 3} & 0_{3\times 6} & -C_{b}^{n}(\omega_{eb}^{b}\times ) & a^{n}\\ 0_{3\times 3} & 0_{3\times 3} & I_{3\times 3} & 0_{3\times 6} & -M_{pv}C_{b}^{n} & M_{pv}v^{n} \end{array}\right],V=\left[\begin{array}{c} V_{v}\\ V_{p} \end{array}\right]$$

$w_{k}$ should satisfy the following conditions:

(7) $$\begin{array}{l} E\{V_{k}\}=0\\ E\{V_{k}V_{i}^{T}\}=\left\{ \begin{array}{l} R_{k}\;i=k\\ 0\;\;\;i\neq k \end{array}\right. \end{array}$$

where $R_{k}$ is the covariance matrix of measurement noise. 

The state prediction and state prediction covariance [26] are given by:

(8) $$\left\{ \begin{array}{l} X_{k}^{-}=\Phi_{k.k-1}X_{k-1}^{+}\\ P_{k}^{-}=\Phi_{k.k-1}P_{k-1}^{+}\Phi_{k,k-1}^{T}+Q_{k-1} \end{array}\right.$$

The measurement update step [26] is provided as:

(9) $$\left\{ \begin{array}{l} K_{k}={\hat{P}}_{k}^{-}H_{k}^{T}{\left(H_{k}{\hat{P}}_{k}^{-}H_{k}^{T}+R_{k}\right)}^{-1}\\ {\hat{X}}_{k}={\hat{X}}_{k}^{-}+K_{k}(Z_{k}-H_{k}{\hat{X}}_{k}^{-})\\ {\hat{P}}_{k}=(I-K_{k}H_{k}){\hat{P}}_{k}^{-} \end{array}\right.$$

where $K_{k}$ is the Kalman gain matrix; the symbols “^” and “~” above a variable represent an estimate and a measurement, while the superscripts “−” and “+” represent the a priori and a posteriori estimates, respectively.

## 3. Optimal RBF Neural Network Aided Robust Kalman Filter

### 3.1. Fault Detection Based on Mahalanobis Distance

Reliable GNSS observation is a prerequisite to achieve high-accuracy with a GNSS/INS integrated navigation system. In ideal conditions, the filtering observation should be Gaussian distributed, so the standard EKF is carried out according to Equations (8)–(9). The square of the Mahalanobis distance [27] from observation $Z_{k}$ to its mean ${\hat{Y}}_{k}^{-}$ is treated as the relevant test statistic witch can be described as:

(10) $$\begin{array}{ll} \gamma_{k} & =M_{k}^{2}={\left(\sqrt{{\left(Z_{k}-{\hat{Y}}_{k}^{-}\right)}^{T}{\left({\hat{P}}_{{\hat{Y}}_{k}}^{-}\right)}^{-1}(Z_{k}-{\hat{Y}}_{k}^{-})}\right)}^{2}\\ & ={\left(Z_{k}-{\hat{Y}}_{k}^{-}\right)}^{T}{\left({\hat{P}}_{{\hat{Y}}_{k}^{-}}^{-}\right)}^{-1}(Z_{k}-{\hat{Y}}_{k}^{-}) \end{array}$$

where $M_{k}^{}$ is the Mahalanobis distance, ${\hat{Y}}_{k}^{-}$ is the observation prediction vector stated above with ${\hat{P}}_{Y_{k}}^{-}$ as its associate covariance matrix which can be written as follows:

(11) $${\hat{Y}}_{k}^{-}=H_{k}{\hat{X}}_{k}^{-}$$

(12) $${\bar{P}}_{{\hat{Y}}_{k}^{-}}^{-}=H_{k}{\hat{P}}_{k}^{-}H_{k}^{T}+R_{k}$$

If dynamical model noise $w_{k-1}$ and observation error are both Gaussian distribution, this test statistic $\gamma_{k}$ should meet Chi-square distributed with degree of freedom *m*:

(13) $$\gamma_{k}\sim \chi_{a}^{2}(m)$$

where *m* is the degree of freedom, i.e., the dimension of the observation, $\chi_{a}^{2}(m)$ is the threshold at significance level *α*. *α* is a small value, in this contribution 1% is adopted. If the actual judging index ${\tilde{\gamma }}_{k}$ is greater than α-quantile, some kinds of observation errors can be thought to exist. 

### 3.2. Robust Kalman Filter

In this paper, a robust EKF with a scaling factor $\lambda_{k}$ is introduced to address the observation gross errors. The observation noise covariance $R_{k}$ can be adapted as:

(14) $${\bar{R}}_{k}=\lambda_{k}R_{k}$$

Then we have:

(15) $$\bar{\gamma }=n_{k}^{T}{\left({\bar{P}}_{{\hat{Y}}_{k}^{-}}^{-}\right)}^{-1}n_{k}=n_{k}^{T}{\left(H_{k}P_{k}^{-}H_{k}^{T}+{\bar{R}}_{k}\right)}^{-1}n_{k}=\chi_{\alpha }$$

so the following equation can be satisfied:

(16) $$f(\lambda_{k})=n_{k}^{T}{\left(H_{k}P_{k}^{-}H_{k}^{T}+{\bar{R}}_{k}\right)}^{-1}n_{k}-\chi_{\alpha }=0$$

Equation (16) is nonlinear in $\lambda_{k}$, so it can be solved iteratively using Newton’s method (we omitted the derivation process):

(17) $$\lambda_{k}(i+1)=\lambda_{k}(i)-\frac{f(\lambda_{k}(i))}{f^{\prime }(\lambda_{k}(i))}$$

(18) $$\left\{ \begin{array}{ll} \lambda_{k}(i)=1 & i=0\\ \lambda_{k}(i+1)=\lambda_{k}(i)-\frac{{\bar{\gamma }}_{k}(i)-\chi_{\alpha }}{n_{k}^{T}{\left({\bar{P}}_{{\hat{Y}}_{k}^{-}}^{-}(i)\right)}^{-1}R_{k}{\left({\bar{P}}_{{\hat{Y}}_{k}^{-}}^{-}(i)\right)}^{-1}n_{k}} & i\geq 1 \end{array}\right.$$

In this paper, the *α*-quantile $\chi_{\alpha }$ of the Chi-square distribution is predetermined, e.g., that for the 6-degree-of-freedom Chi-square distribution with the significance level being 1%, it is 16.812.

As we can see from Equation (8), an inflated covariance of the observation noise will result in an inflated covariance of the observation prediction. So another scalar factor, used to adjust the covariance of the observation prediction, can also be introduced to ensure the robustness as follows:

(19) $${\bar{P}}_{{\hat{Y}}_{k}^{-}}^{-}=\kappa_{k}P_{{\hat{Y}}_{k}^{-}}^{}$$

and $\kappa_{k}$ can be calculated analytically with:

(20) $$\kappa_{k}=\left\{ \begin{array}{lll} 1 & \mathrm{if} & {\tilde{\gamma }}_{k}\leq \chi_{\alpha }\\ \frac{{\tilde{\gamma }}_{k}}{\chi_{\alpha }} & \mathrm{else} & \end{array}\right.$$

The robust Kalman gain matrix changes to:

(21) $$K_{k}=\kappa_{k}{\hat{P}}_{k}^{-}H_{k}^{T}{\left(\kappa_{k}H_{k}{\hat{P}}_{k}^{-}H_{k}^{T}+\lambda_{k}R_{k}\right)}^{-1}$$

Through this method, the actual observation is less weighted and the information from the dynamic model is more weighted. The effect of the actual observation gross errors is effectively resisted.

### 3.3. Adaptive Kalman Filter

Even if the observation is reliable, it still requires more accurate dynamic model for both INS and GPS errors, since it is usually difficult to set a certain stochastic model for each inertial sensor that works efficiently in all environments [28]. In a low-cost GNSS/MINS integrated system, inertial sensor also includes gross error due to unmeasurable external disturbances and high dynamics which against stochastic model, and may be harmful for state prediction vector and its covariance [6]. So an adaptive Kalman filter based on state prediction covariance should be constructed to adjust the contribution of the predicted states from the IMU sensors. 

A conventional adaptive Kalman filter [29] is given by:

(22) $$P_{k}^{-}=(\Phi_{k,k-1}P_{k-1}^{+}\Phi_{k,k-1}^{T}+Q_{k-1})/\alpha_{k}$$

where $\alpha_{k}$ is adaptive factor, $\alpha_{k}$ should less than 1 when there’s large dynamical model error in state prediction.

The fading factor filtering established by Xia [29] has the distinct advantage that it remains convergent and tends to be optimal in the presence of model errors. Therefore we bring it into Equation (22) as an adaptive factor:

(23) $$\alpha_{k}=\frac{1}{\lambda_{k}}=\frac{1}{\max\left\{1,\frac{\mathrm{tr}[N_{k}]}{\mathrm{tr}\left[M_{k}\right]}\right\}}$$

where tr is the trace of a matrix:

(24) $$M_{k}=H_{k}\Phi_{k.k-1}{\hat{P}}_{k}^{-}\Phi_{k.k-1}^{T}H_{k}^{T}$$

(25) $$N_{k}=C_{0k}-R-H_{k}Q_{k}H_{k}^{T}$$

(26) $$C_{0k}=\{\begin{array}{c} \frac{\lambda_{k}{\bar{v}}_{k}{\bar{v}}_{k}^{T}}{1+\lambda_{k}},\;\;k>1\\ \frac{1}{2}{\bar{v}}_{0}{\bar{v}}_{0}^{T},\;\;k=1 \end{array}$$

where ${\bar{v}}_{k}=Z_{k}-H_{k}{\hat{X}}_{k}^{-}$ is residual sequence.

By this way, the availability factor of historical states information has been reduced, in other words, the availability factor measurement information at present time has been increased. The effect of the dynamic model errors is therefore effectively resisted.

### 3.4. Radial Basis Function Neural Network Algorithm

**Figure 1:**
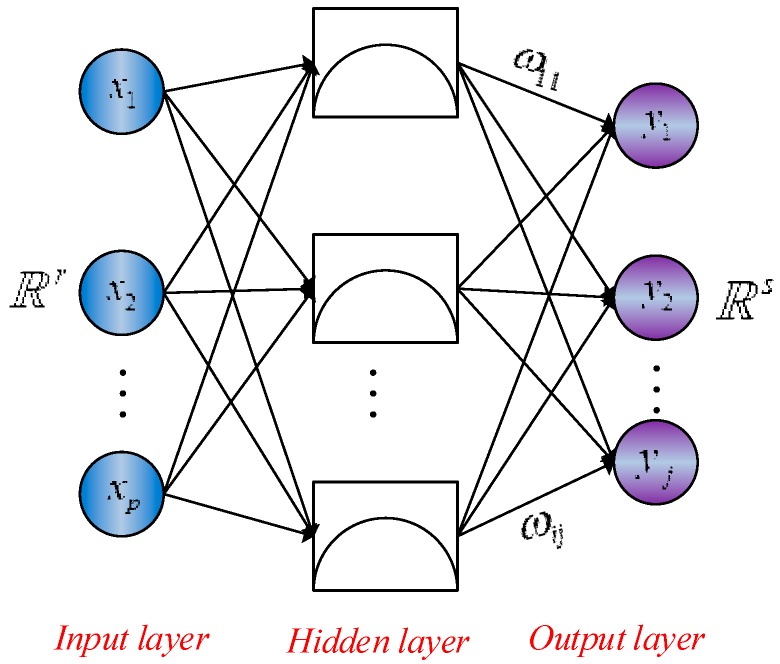
The architecture of RBF neural network.

RBF neural network is a popular kernel function used in various kernelized learning algorithms. It typically has three layers: an input layer, a hidden layer with a non-linear RBF activation function and a linear output layer [30,31]. The architecture of RBF neural network is shown in Figure 1, which can be considered as a mapping $R^{r}\to {\mathbb{R}}^{s}$.

All inputs are connected to each hidden neuron (neural unit). Then a radial basis function is used as the activation function in the hidden neuron. Usually, the Gaussian function is preferred among all possible radial basis functions due to the fact that it is factorizable. Consequently, the norm is typically taken to be the Euclidean distance and the radial basis function is commonly taken to be Gaussian. The further distance neuron’s input gets from the center of radial basis function, the lower level of the activation for the neurons become. Let $x_{p}\in {\mathbb{R}}^{r}$ be the input vector, the radial basis function is provided as [32]:

(27) $$R(x_{p}-c_{i})=\exp(-\frac{1}{2\sigma^{2}}{\left\|x_{p}-c_{i}\right\|}^{2})$$

where *P* is the total number of samples, $x_{p}={\left(x_{1}^{p},x_{2}^{p},\ldots ,x_{m}^{p}\right)}^{T}$ is *p*th input sample; $\left\|x_{p}-c_{i}\right\|$ represents Euclidean norm; $c_{i}$ is the center of the Gaussian function, σ is the variance of the Gaussian function, respectively. Then the output of the network is denoted as:

(28) $$y_{j}=\sum_{i=1}^{h}\omega_{ij}\exp(-\frac{1}{2\sigma^{2}}{\left\|x_{p}-c_{i}\right\|}^{2})\;\;\;j=1,2\ldots ,n$$

where $\omega_{ij}$ is the link weight from the hidden layer to the output layer; i=1,2,3,…,h is the number of neurons in the hidden layer; $y_{j}$ is the network’s actual output of the *j*th node corresponding to the input sample.

It is assumed that *d* is the desired output of the sample, so the variance of the odd function is given by:

(29) $$\sigma =\frac{1}{P}\sum_{j}^{m}{\left\|d_{j}-y_{i}c_{i}\right\|}^{2}$$

The RBF learning algorithm can be expressed as follows:

Step 1: The center of the basis function $c_{i}$ is calculated with K-means clustering.

(1) Network initialization. *h* training samples are randomly selected as the center of clustering $c_{i}$, in other words, the center of the Gaussian function.
(2) Grouping. The Euclidean distance from the mean $c_{i}$ to $x_{p}$ is computed, then the training samples are put into each clustering class $\vartheta_{p}$ (p=1,2,3,…,P) based on nearest neighbor rule.
(3) Adjusting the center of clustering. The mean value of training samples are computed in each clustering class $\vartheta_{p}$, i.e., the new center of clustering $c_{i}$. If the new $c_{i}$ gets no more change, then $c_{i}$ finally become the center of radial basis function. But if not, go to step 2 to recompute.

Step 2: Computing the variance of the Gaussian function $\sigma_{i}$.

The variance of the Gaussian function $\sigma_{i}$ can be satisfied by the expression:

(30) $$\sigma_{i}=\frac{c_{\max}}{\sqrt{2h}}i=1,2,\ldots ,h$$

where $c_{\max}$ represents furthest Euclidean distance in every $c_{i}$.

Step 3: Computing the link weight of the neural unit between the hidden layer and the output layer, which is given by:

(31) $$\omega =\exp(\frac{h}{c_{\max}^{2}}{\left\|x_{p}-c_{i}\right\|}^{2})\;\;\;i=1,2,\ldots ,h;\;p=1,2,\;\ldots P$$

### 3.5. Optimal RBF Neural Network Aided Navigation Fault Recognition

We take the integral of raw acceleration $f_{ib}^{b}$ and rotation rates $\omega_{ib}^{b}$ as training inputs of RBF algorithm, which considered as the summation of angle and velocity increments [17]. Thereafter, we take GPS/BDS position difference as training outputs, which is a three dimensional vector with north, east and down position as components. The expression can be written as:

(32) $$\overset{k}{\underset{n=1}{FN_{RBF}}}(\mathrm{str},\int_{t-1}^{t}f_{ib}^{b},\int_{t-1}^{t}\omega_{ib}^{b})=\Delta \bar{P}={\left[\begin{array}{ccc} \Delta B & \Delta L & \Delta H \end{array}\right]}^{T}\left[\begin{array}{c} R_{M}\\ \cos(B)R_{N}\\ -1 \end{array}\right]$$

where str is the RBF network structure; $f_{ib}^{b}$ is the raw acceleration of the accelerometer; $\omega_{ib}^{b}$ is the rotation rate of the gyro; $\Delta \bar{P}$ is the position increments in the n-frame; ΔB, ΔL and ΔH are the differences of geodetic coordinates, respectively; $R_{M}$ is the meridian radius of curvature; $R_{N}$ is the transverse radius of curvature [33].

**Figure 2:**
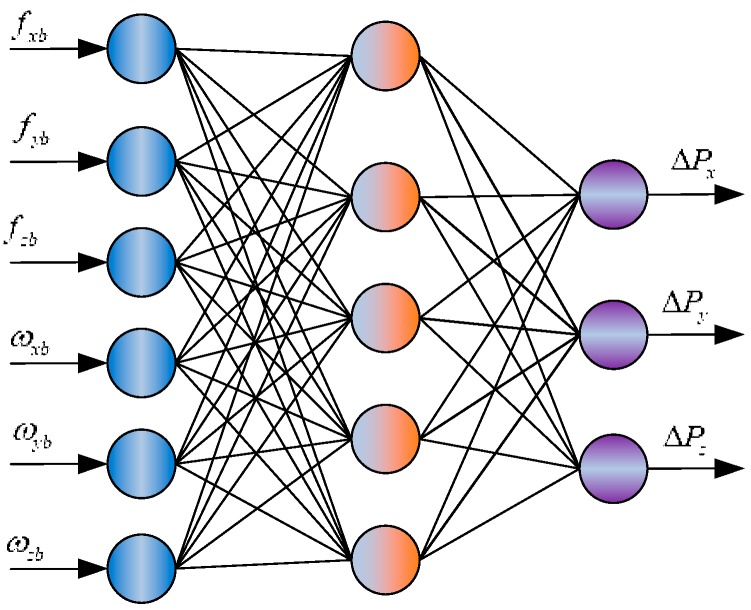
Input and output of a radial basis function network.

As shown in Figure 2, it’s a nonlinear problem from IMU outputs to position changes. The output of the network may predict the optimal estimation of state parameters of dynamic model, provided that differential navigation solution is reliable and network learning is reasonable before k epoch, so a reliable observation is a key precondition to obtain optimal estimation. For this purpose, we propose the following strategies to improve the quality of observation information to obtain optimal RBF (ORBF) training results:

Step 1: GPS/BDS abnormal observations are eliminated firstly.

The standard deviation of residual sequence is given by:

(33) $$\delta_{V_{k_{i}}}^{0}=\mathrm{median}\left\{\left|V_{ki}\right|\right\}/0.6745$$

If $\left|V_{ki}\right|>c\delta_{V_{k_{i}}}^{0}$, both the GPS/BDS observation and corresponding residual sequence should be removed, where *c* is a constant which may be determined by 2.0 [11].

Step 2: Optimal spread factor of RBF network is adjusted online during integrated system dynamic initialization phase. 

The root mean square (RMS) of RBF predicted result can be written as:

(34) $${\mathrm{RMS}}_{pre}=\sqrt{\frac{1}{N}\sum_{k=1}^{N}{\left(Z_{\mathrm{GNSS}}-Z_{\mathrm{pre}}\right)}^{2}}$$

where $Z_{\mathrm{GNSS}}$ is GNSS observation during training phase, $Z_{\mathrm{pre}}$ is predicted value of the network, *N* is the number of epochs. Then the network structure is trained by the following function, which can be described as:

(35) $$\mathrm{str}=\mathrm{newrbe}(\int f_{ib}^{b},\int \omega_{ib}^{b},Z_{\mathrm{GNSS}},s)$$

where newrbe represents RBFNN function, *s* is the spread factor of the network. After the network structure is determined, according to (32), the RBFNN output can be satisfied:

(36) $$Z_{pre}=\overset{k}{\underset{n=1}{FN_{RBF}}}(\mathrm{str},\int_{t-1}^{t}f_{ib}^{b},\int_{t-1}^{t}\omega_{ib}^{b})$$

Optimal spread factor will make sure of getting the optimal output. From our experience, the initial value of spread factor *s* is set at 0.5 and the maximum value is limited to 10. It can be solved iteratively by Equations (34)–(36) with following criterion:

(37) $${\mathrm{RMS}}_{\mathrm{pre}}=\min$$

Step 3: The RBF network training is carried out with the rest of observation and optimal spread factor by sliding window method. 

In consideration of the bias instability of low-cost IMU will change over time, in this research, the width of the sliding window is set at 50. By this way, the RBF neural network can be more efficient, reliable and stable after the preprocessing above is applied on the network inputs. The predicted position with optimal RBF can avoid effect of probably fault or abnormal information at *k* epoch and be used to detect and locate the current observation information. That is to say, the source of system failure can be distinguished by following decision threshold and robust or adaptive Kalman filtering is automatically chosen for the integrated system:

$$\left|Z_{\mathrm{pre}}-Z_{\mathrm{GNSS}}\right|\geq 3{\mathrm{RMS}}_{\mathrm{pre}}\to \mathrm{robust}\;\mathrm{Kalman}\;\mathrm{filtering}$$

$$\left|Z_{\mathrm{pre}}-Z_{\mathrm{GNSS}}\right|<3{\mathrm{RMS}}_{\mathrm{pre}}\to \mathrm{adaptive}\;\mathrm{Kalman}\;\mathrm{filtering}$$

**Figure 3:**
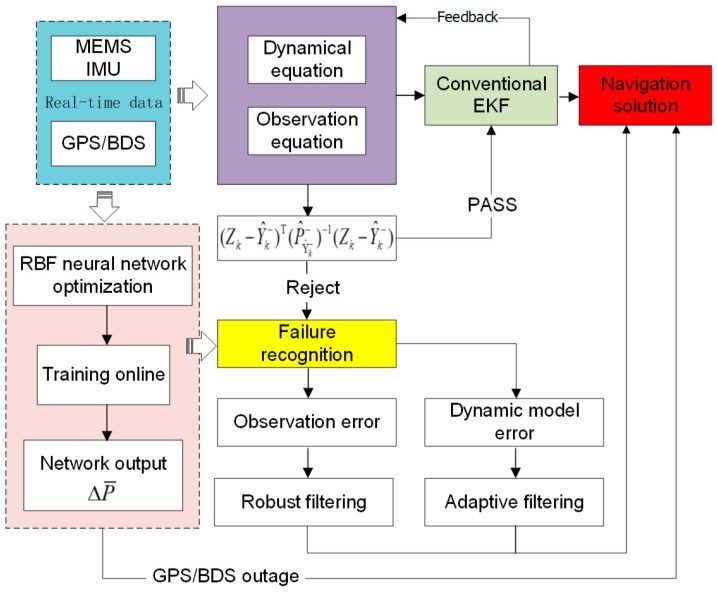
Technical scheme used in this research.

Furthermore, during satellite signals outage periods, the ORBF aided intelligent navigation system architecture provides prediction of position differences that can also replace the Kalman filter for an intelligent navigation information support. The technical scheme of this paper is shown in Figure 3.

## 4. Field Test and Performance Evaluation

### 4.1. Field Test Details

In order to evaluate the performance of the proposed method, a field test based on a GPS/BDS RTK/MEMS-IMU integrated system was conducted around the campus of China University of Mining and Technology (CUMT). A Trimble receiver was fixed as a reference station on the campus. During the test, the data from GPS and BDS dual constellation were used for analysis with a sampling rate of 1 Hz. 

**Figure 4:**
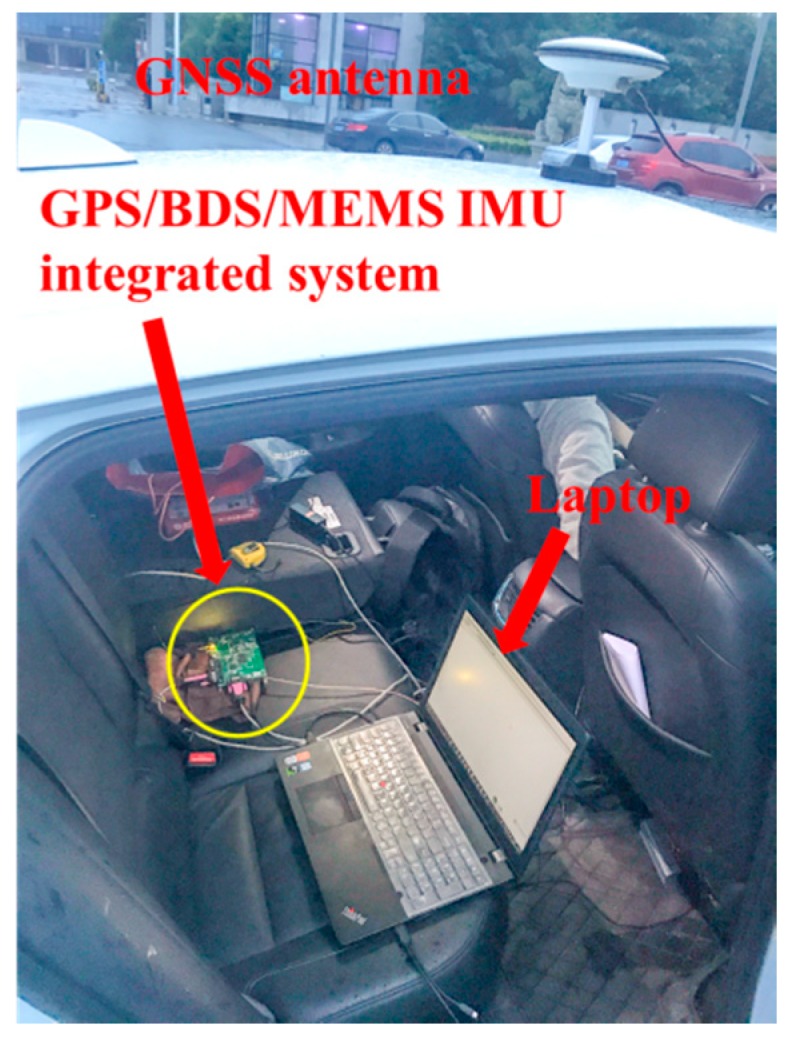
Hardware system configuration.

In the meantime, a GNSS/MEMS-IMU integrated navigation system with a SCC2230-E02 IMU and Unicorecomm-UB380 board as rover station with its Novatai antenna was used to perform the field test above the roof of the test vehicle. 1 Hz GPS/BDS carrier phase and pseudo-range data and 100 Hz INS raw data were received and stored in a laptop for post-processing. The hardware system configuration of the rover station is shown in Figure 4.

The specifications for the low-cost MEMS IMU are given in Table 1. In addition, a magnetometer also be integrated into this system to help initialize the heading angle. The initial attitude for the IMU is given in Table 2. 

**Figure 5:**
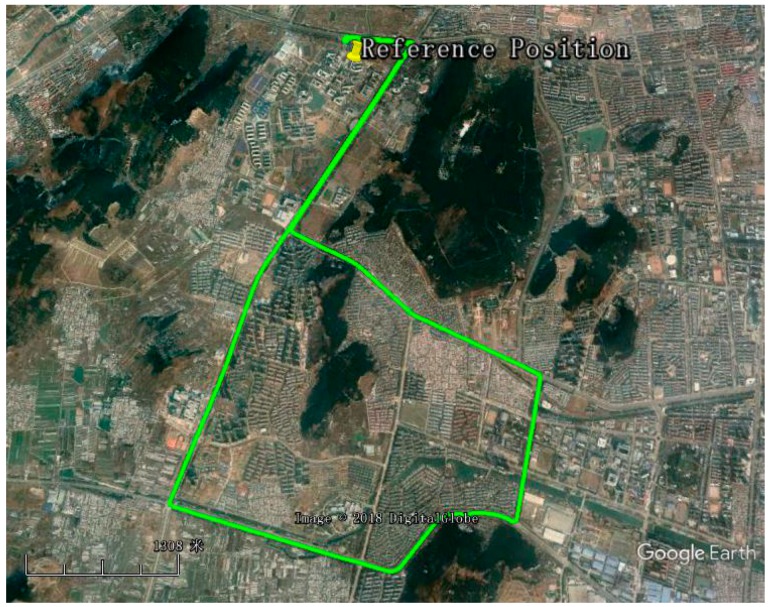
Ground track of field test.

The trajectory obtained with the standard Real Time Kinematic (RTK) algorithm by the modified GPStk software is given in Figure 5.

**Figure 6:**
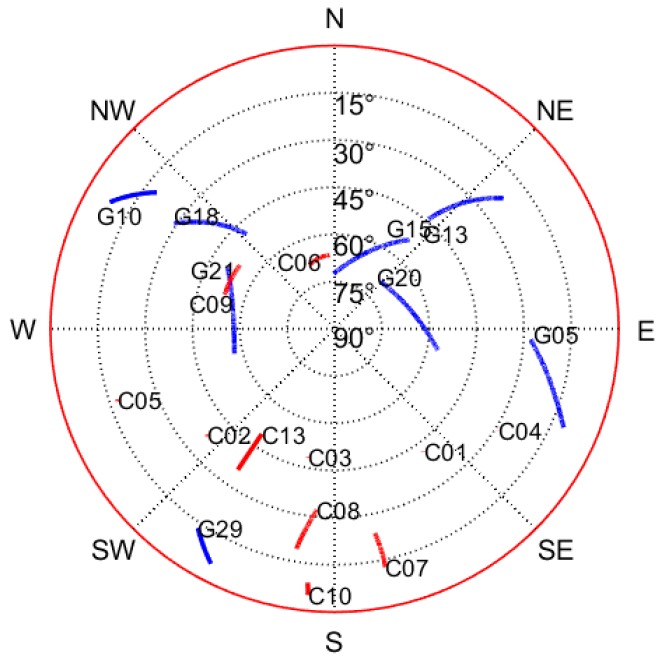
Satellites visibility in the test.

A total of 3005 s GPS/BDS data were collected in this test in 10 November 2017, which started from GPS time 290,633 s and ended at 293,638 s. The observed satellites at the rover station are shown in Figure 6, which shows the visibility of GPS and BDS satellites.

**Figure 7:**
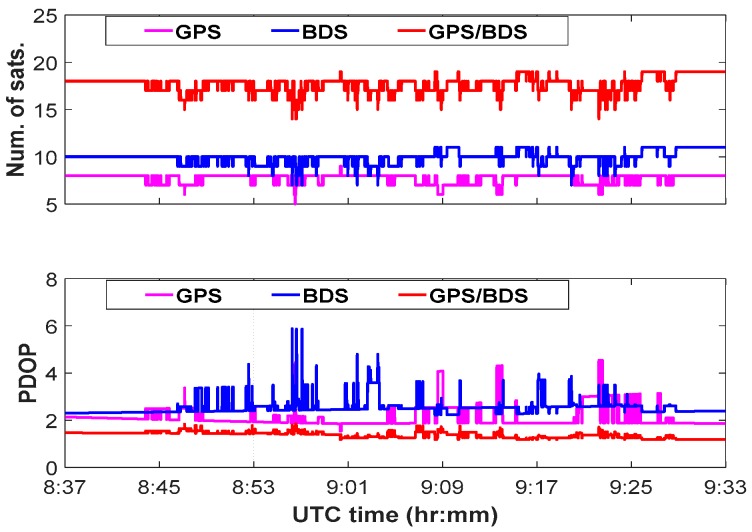
Satellites visibility in the test.

Figure 7 plots the number of visible satellites and the position dilution of precision (PDOP) variations for the combined GPS/BDS system in the test. The average PDOPs for the combined system can reach at the level of less than 1.5, which was evidently better than the individual system. There are no observation outliers in the GNSS observations during the period of the vehicle test.

**Figure 8:**
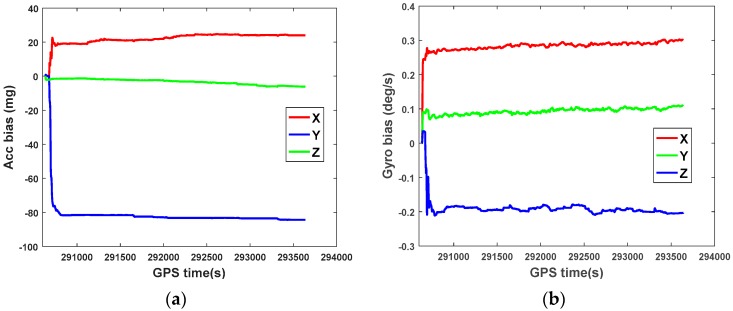
Accelerometer bias (**a**) and gyro bias (**b**) for IMU sensors.

A loosely-coupled strategy is adopted to calculate the navigation solutions of the GPS/BDS/INS integrated system based on conventional EKF. Figure 8 illustrates the estimation of the accelerometer bias and the gyro bias for IMU sensors in integrated system, as expected, the sensor bias quickly converges to a stable value after the initial disturbances and the estimated sensor bias is consistent with the sensor specifications provided by the manufacturer.

### 4.2. Optimal RBF Neural Network Training

If GPS/BDS signals are available, the standard EKF strategy is adopted to calculate the navigation solutions of the GPS/BDS/INS integrated system. Simultaneously, the specific increments of the GPS/BDS position are trained based on the ORBF neural network with corresponding acceleration and angular rate increments of the INS measurements as the input. 

**Figure 9:**
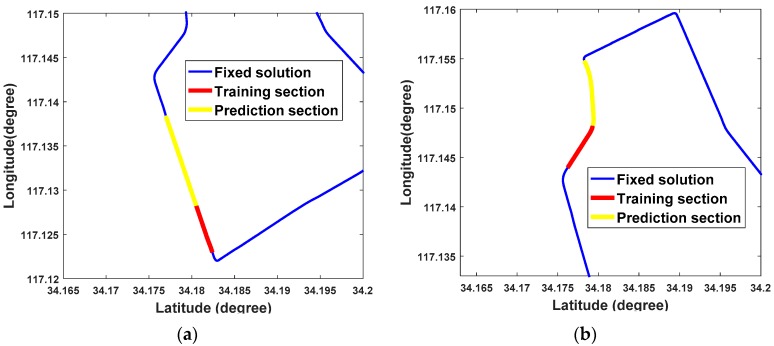
The corresponding route in Test 1 trajectory (**a**) and Test 2 trajectory (**b**).

**Figure 10:**
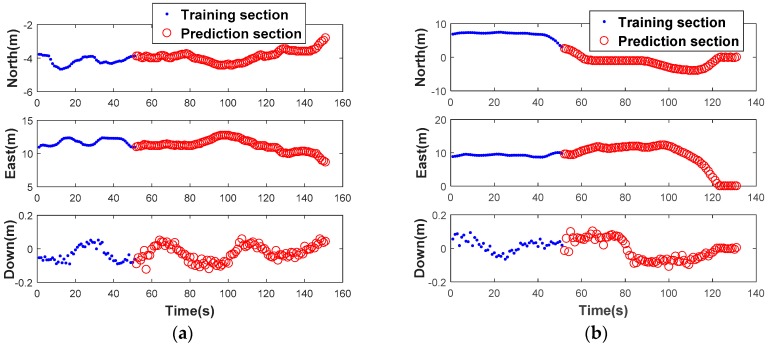
GPS/BDS Position increments in Test 1 (**a**) and Test 2 (**b**).

The feasibility of the ORBF neural network is verified using two trajectories which can be seen in Figure 9. For the authenticity of the test, one chosen route is a straight line and another one is a curve. The GPS/BDS position increments of the two routes produced by highly precise double-differenced (DD) carrier measurements are shown in Figure 10.

*Test 1*: Navigation solutions between the 1481th and 1580th seconds are provided when the vehicle moved along a straight line, 50 groups of data from the 1430th to 1480th seconds were chosen as the RBF training samples. 

*Test 2*: Navigation solutions between the 1701th and 1780th seconds are provided when the vehicle moved along a curve, 50 groups of data from the 1650th to 1700th seconds were chosen as the RBF training samples as well. 

**Figure 11:**
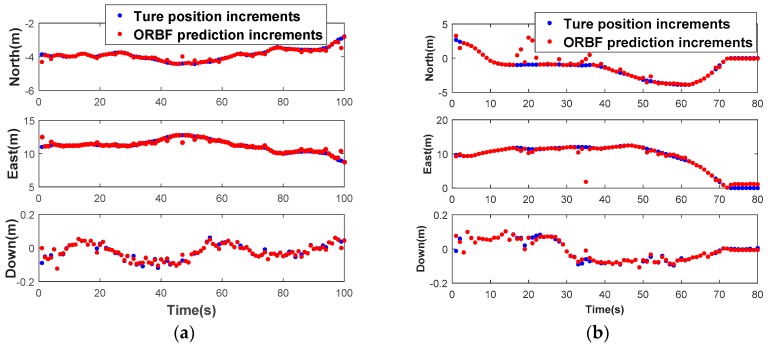
Prediction performance with ORBFNN algorithm in Test 1 (**a**) and Test 2 (**b**).

**Figure 12:**
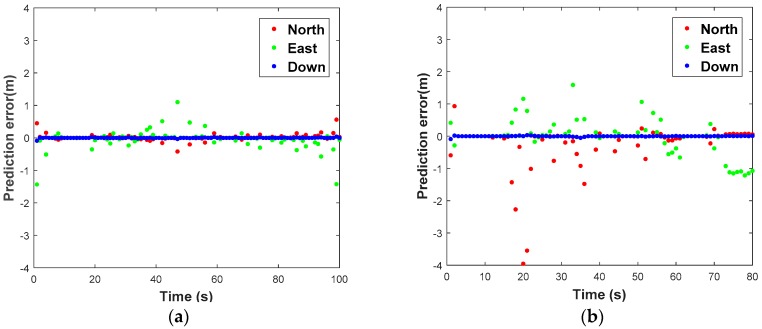
Prediction error with ORBF network algorithm in Test 1 (**a**) and Test 2 (**b**).

What needs to be explained in advance is that the GPS/BDS observation has been preprocessed with the strategy mentioned in Section 3.3. In the interest of saving space, we omit discussing this step and the optimal spread factor is given directly i.e., 0.75 in our actual test. The prediction performance with ORBF network algorithm of the two tests is illustrated in Figure 11. To clarify the performance further, the prediction error of the two tests in n- frame is shown in Figure 12. The RMS error and prediction failure rate (≥3RMS) of the prediction errors of the two tests are summarized in Table 3.

As can be seen from the Table 3, the prediction error is significantly less than 0.5 m in test 1, and less than 1 m in the other test 2 if we take no account of several prediction gross errors. Hence, the accuracy of the predicted position in test 1 is obviously much higher than that in test 2 due to route type and smooth operation. The average RMS values are 0.099 m, 0.257 m and 0.012 m for north, east and down components respectively in test 1, 0.773 m, 0.590 m and 0.013 m for north, east and down components in test 2. The predicted anomaly is within a controllable range, which less than 7.5% in both of the test.

It is concluded that ORBF algorithm proposed in this study is able to predict GNSS position increments with sub-meter level precision, but due to biases, random walk error and stochastic noise of the low-cost IMU sensors, it will affect the high-dynamic position increments prediction. A high precision IMU may perform better owing to its low-noise characteristics.

### 4.3. Performance of the Proposed Method

**Figure 13:**
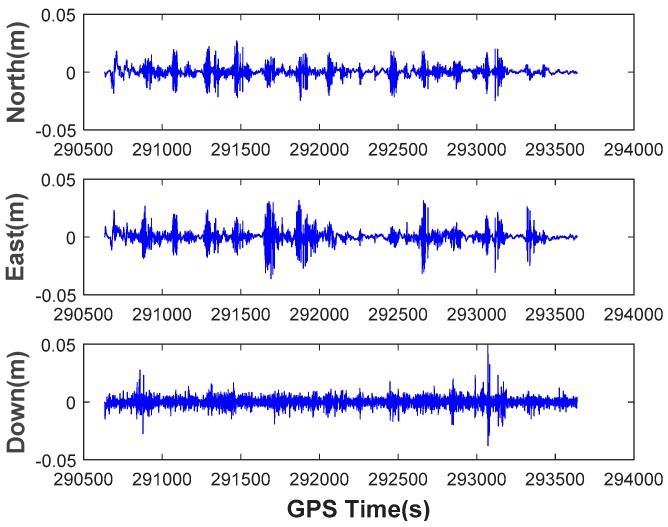
Position error of the integrated navigation system.

By using the conventional EKF model, we obtained the estimated position error. The highly precise results from double-differenced (DD) carrier measurements are used only as “true values” for comparing with the results from the integrated measurements. Figure 13 plot the position error of the integrated system respect to the GPS/BDS RTK reference solution when using standard EKF model.

As can be seen from Figure 13, centimeter-level positioning accuracy is achievable through the GPS/BDS/INS integrated system after the dynamic initial alignment finished. In most cases, the accuracy is dramatically better than 3 cm. Then we simulate a specific complex environment to demonstrate the priorities of the proposed integration scheme. The complex urban areas environment can be described as:

(1) 1 m, 2 m, 3 m, 4 m, 5 m, 10 m outliers, 6 in total, were intentionally given to the carrier phase measurement every other 200 epochs from 600th to 1600th epoch.
(2) 2.8 g, 2.4 g, 2 g, 1.6 g, 1.2 g 0.8 g, 0.4 g outliers, 7 in total, were intentionally given to the Z-axis of the MEMS IMU measurement every other 200 epochs from 1800th to 3000th epoch.

The following five schemes are examined:

- Scheme 1: standard extended Kalman filter (EKF).
- Scheme 2: robust Kalman filter (RKF).
- Scheme 3: adaptive Kalman filter (AKF).
- Scheme 4: adaptive robust Kalman filter (ARKF).
- Scheme 5: ORBF-aided adaptive robust Kalman filter (RRKF).

**Figure 14:**
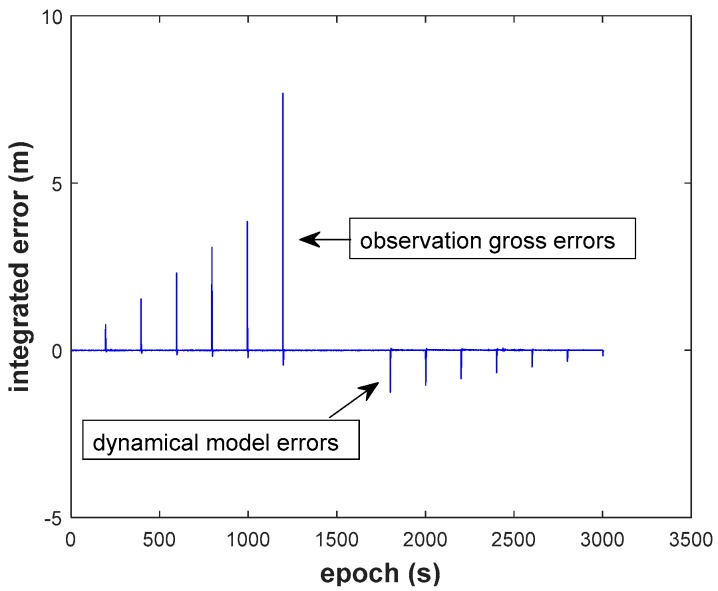
Height position errors of EKF.

**Figure 15:**
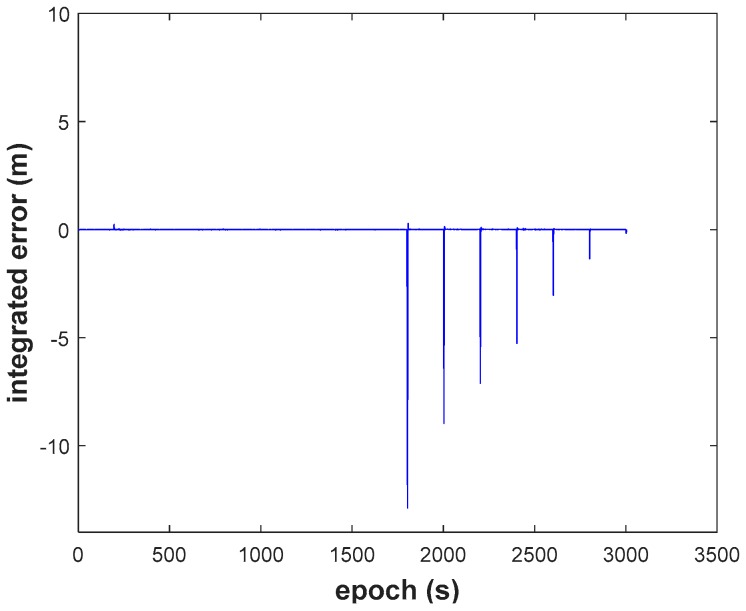
Height position errors of RKF.

**Figure 16:**
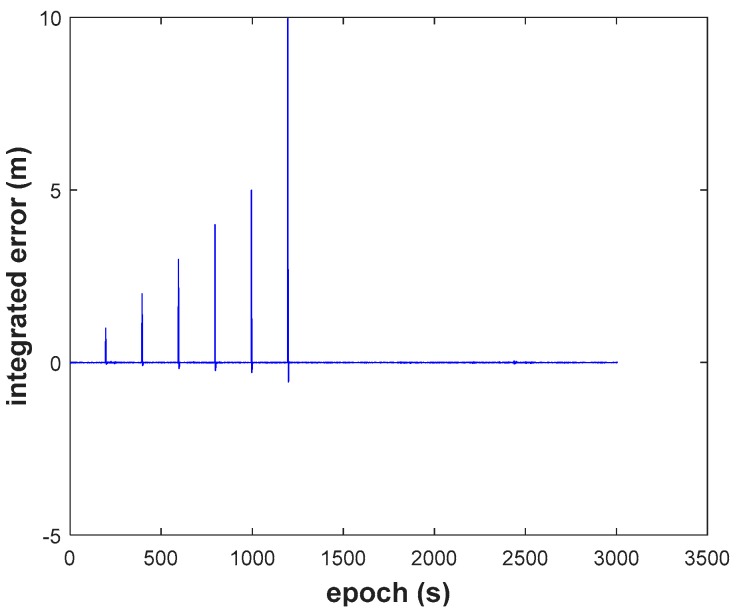
Height position errors of AKF.

**Figure 17:**
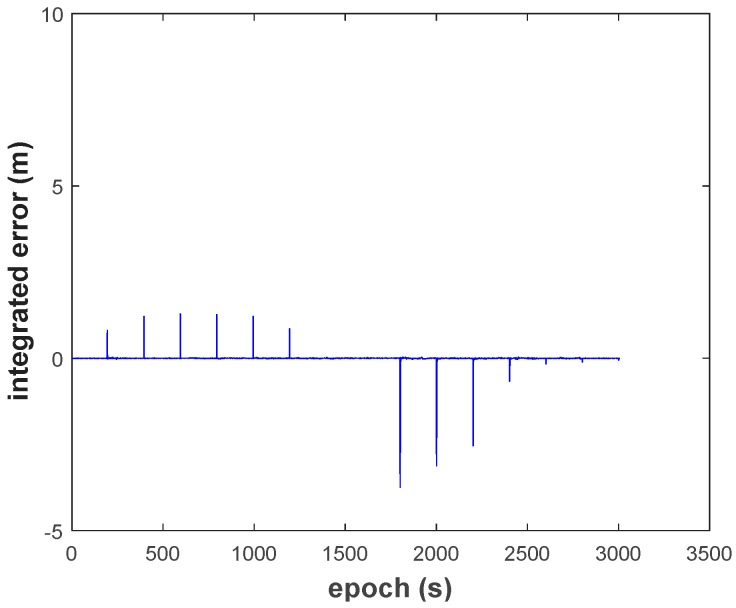
Height position errors of ARKF.

**Figure 18:**
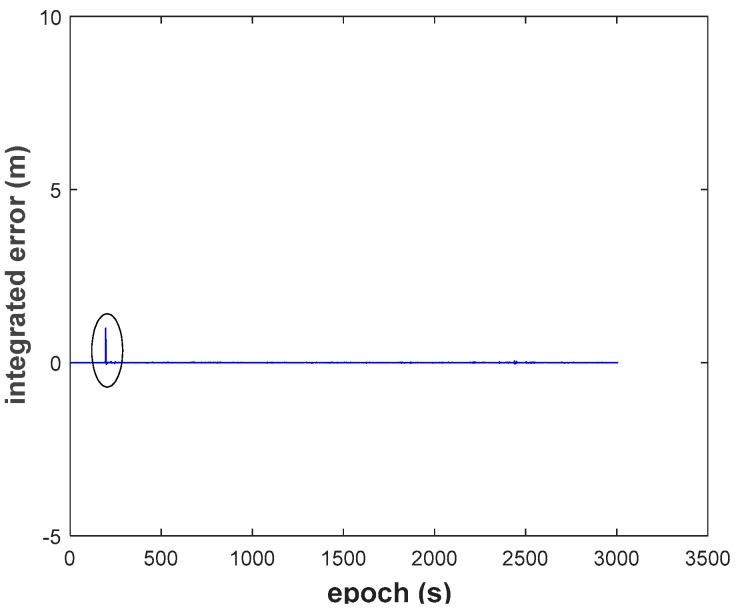
Height position errors of RRKF.

The height position errors are plotted only to verify the algorithm performance. In this test, the size of the training sliding window is set to 50 epochs. From our theoretical derivation, actual test, analysis and comparison, the following conclusions can be drawn:

(1) The two types of errors are obviously reflected in the results of the conventional EKF (Scheme 1, Figure 14). The filtering has no ability to resist the two types of outlier. The maximum integrated error caused by observation gross error reaches to 7.681 m, and the maximum integrated error caused by dynamic model error is −1.247 m.

(2) We recognize that the RKF (Scheme 2, Figure 15) does resist the influence of the GNSS observation gross error, but it cannot resist the influence of the dynamic model error. The filtering performs even worse than EKF when dynamic model errors occur. On the contrary, the AKF (Scheme 3, Figure 16) can balance the contribution of updated parameters and the new measurements, but it needs the support of correctly observation. It should also be noted that AKF cannot resisted the influence of the observation gross errors.

(3) Comparing Schemes 2, 3, to the ARKF algorithm (Scheme 4, Figure 17), we find that ARKF cannot get reasonable results in the case where outliers exist. The ARKF strategy performs unsteadily, sometimes even anomalously due to the fact the two types of errors are treated as the same situation by ignoring characteristics of the errors.

(4) Among the above algorithms, the results from RRKF (Scheme 5, Figure 18) are the best. The two types of errors are effectively detected and identified by the optimal RBF neural network strategy. This algorithm can not only resist the impact of observation gross errors, but also reduce the dynamic model errors in time. This indicates that the approach of integrated navigation information fusion based on the RBF neural network aided Kalman Filter is more effective than the traditional method.

(5) However, as we can see in Figure 18, there’s still an abnormal point marked in the figure even though RRKF performs much better than the other methods. It is caused by the ORBF neural network prediction gross error mentioned in Section 4.2. Speaking frankly, this error-detecting strategy has one shortcoming that it is not sensitive enough to quite small observation gross errors. So the ‘success rate’ of quite small observation outliers identification should be discussed in the actual test latter.

**Figure 19:**
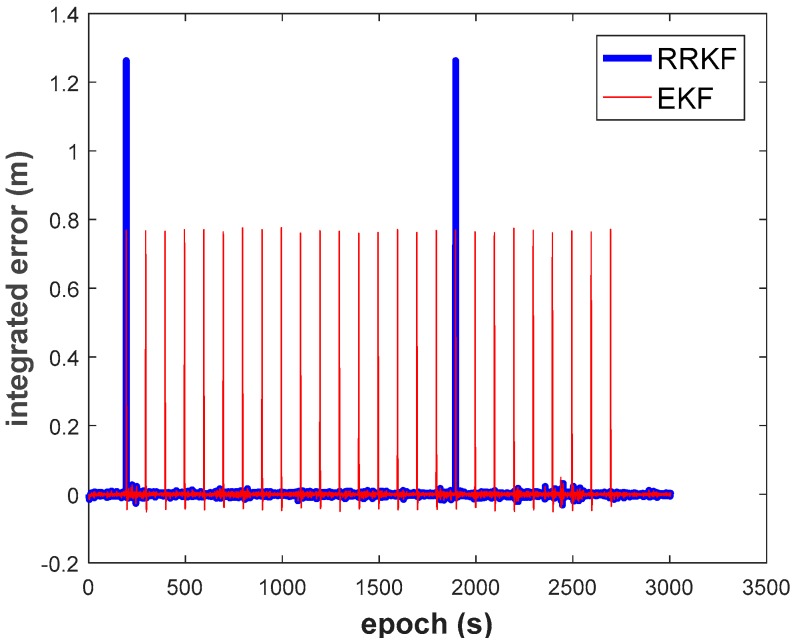
Small dense observation outliers detecting with ORBF strategy.

For the purpose of evaluating the ‘success rate’ of the ORBF neural network strategy, 1 m dense observation gross errors, 25 in total, were intentionally added to the carrier phase measurement every other 50 epochs throughout the whole test process. As the result shown in Figure 19, the reliability of the neural network proposed in this paper achieved was 92% for small observation outliers identification.

**Figure 20:**
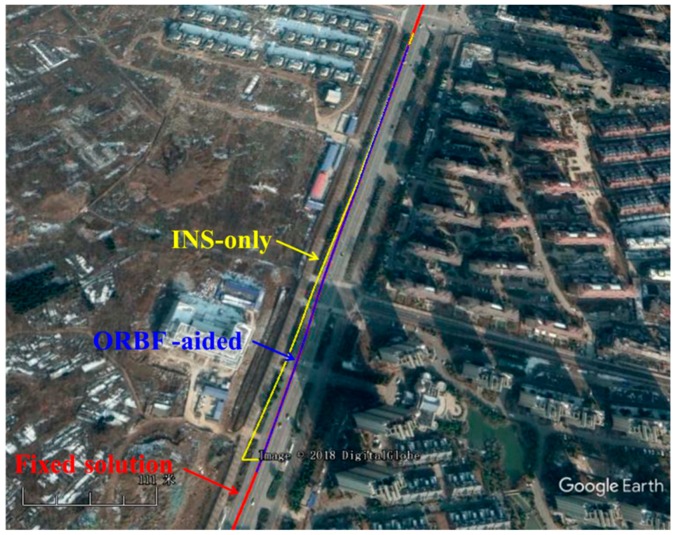
Ground track with three strategies during GPS/BDS outage.

**Figure 21:**
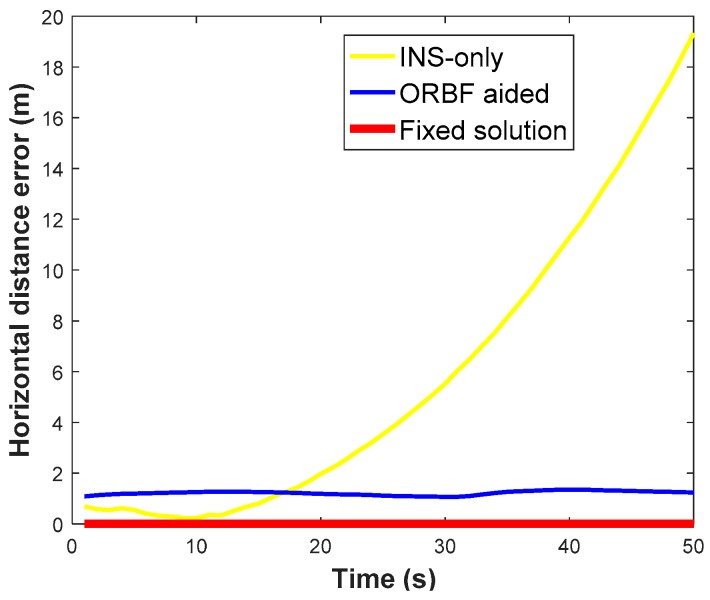
Horizontal distance error of with three strategies during GPS/BDS outage.

Moreover, a GPS/BDS signal outages of 50 s was intentionally introduced to the raw navigation solution to demonstrate the superiorities of the ORBF-aided integrated navigation solution. Three kinds of ground track, which were generated by a standard integrated navigation solution and RBF- aided integrated navigation solution are shown in Figure 20, respectively. The horizontal distance error of the INS-only estimation and ORBF-aided INS estimation are plotted in Figure 21, with the RTK fixed solution as the reference value. 

It is clear that the proposed algorithm provides more accurate results in the absence of GPS/BDS signal. Without neural network aid, the INS position drift error will become larger rapidly with the increase of the outage duration and reaches 19.32 m after 50 s. With the help of the ORBF neural network algorithm, the horizontal distance error can be decreased to about 1 m during the complete GPS/BDS outage.

## 5. Conclusions

In this contribution, we have developed a novel ORBFNN enhanced adaptive robust algorithm and tested it on a vehicle in an urban area. GPS/BDS RTK/IMU real-time integrated navigation experiments were conducted with the proposed algorithm. The comparison of results with some other conventional methodologies for adaptive-robust problems indicates that the proposed ORBFNN-enhanced adaptive robust algorithm performs satisfactorily and is most effective. The highlights of this work are summarized as follows:

(1) The reliability of the combined GPS/BDS system is dramatically improved compared with a single system, and the satellite visibility and DOP are still qualified in urban environments.
(2) An optimal principle to train a classical RBF neural network are proposed, fully considering abnormal observations and the optimal spread of the network. The effect of the network prediction performs well enough to obtain reasonable GNSS position changes.
(3) The GNSS/INS integrated system FDR methodology is firstly proposed and realized by taking the Mahalanobis distance as the fault detection procedure and taking an ORBFNN network prediction as the fault recognition procedure, i.e., to distinguish observation outliers from dynamical model errors in this paper. The system can choose robust or adaptive Kalman filtering autonomously to resist navigation faults in complex urban areas.
(4) The test results indicated that RRKF method can effectively detect, identify, and resist both dynamical model errors and observation gross errors in the integrated system. The performance is superior to traditional methods, and 92% of small observation outliers could be identified in the test.
(5) In addition, we have examined the ORBFNN prediction performance for the low-cost MEMS IMU during GNSS outages, in which the horizontal distance error was less than 1 m during a 50 s GPS/BDS outage in the test.

## Figures and Tables

**Table 1:** MEMS grade IMU technical data.

Parameters	Gyroscope	Accelerometer
Bias	1 deg/s	±16 mg
Scale factor	5000 ppm	500 ppm
Random walk	0.4 deg/sqrt(h)	6 mg/sqrt(Hz)

**Table 2:** Initial attitude.

Attitude	Value
head	−169.003°
pitch	8.278°
roll	0.739°

**Table 3:** Prediction RMS and Failure rate.

	RMS (North)	RMS (East)	RMS (Down)	Failure Rate
Test 1	0.099 m	0.257 m	0.012 m	3%
Test 2	0.733 m	0.590 m	0.013 m	7.5%
